# Ropivacaine with intraspinal administration alleviates preeclampsia-induced kidney injury via glycocalyx /alpha 7 nicotinic acetylcholine receptor pathway

**DOI:** 10.1080/21655979.2022.2080365

**Published:** 2022-05-29

**Authors:** Shen Sun, Yaojun Lu, Fubo Tian, Shaoqiang Huang

**Affiliations:** Department of Anaesthesiology, Obstetrics and Gynaecology Hospital of Fudan University, Shanghai, China

**Keywords:** Preeclampsia, ropivacaine, intraspinal injection, kidney injury, glycocalyx, α7nAChR

## Abstract

Preeclampsia is characterized by hypertension and proteinuria, which is associated with kidney injury. Glycocalyx (GCX) degradation mediated endothelial injury can result in proteinuria and kidney damage. alpha 7 nicotinic acetylcholine receptor (α7nAChR) connects nervous and immune systems to respond to stress or injury. We aimed to explore the protective effect and mechanism of intraspinal analgesia on maternal kidney injury in preeclampsia. Endotoxin-induced preeclampsia rats treated with ropivacaine via intraspinal administration. Renal histopathological examination was performed, cell apoptosis in the kidney, the levels of Glycocalyx markers of Syndecan-1 and heparin sulfate (HS) in maternal serum, Syndecan-1 along with α7nAChR in the kidney were measured. Our results showed that kidney injury was obviously in preeclampsia rats with proteinuria, endothelial damage, higher apoptosis rate, increasing levels of Syndecan-1 and HS in serum, upregulated Syndecan-1 expression but downregulated α7nAChR expression in kidney. Preeclampsia rats treated with intraspinal injected ropivacaine attenuated preeclampsia-induced kidney injury as Syndecan-1 and HS were decreased in serum, Syndecan-1 expression was suppressed as well as α7nAChR was activated in the kidney. Our results suggested that Ropivacaine administered through the spinal canal may protect preeclampsia-induced renal injury by decreasing GCX and α7nAChR activation.

## Graphical Abstract

**Figure:**
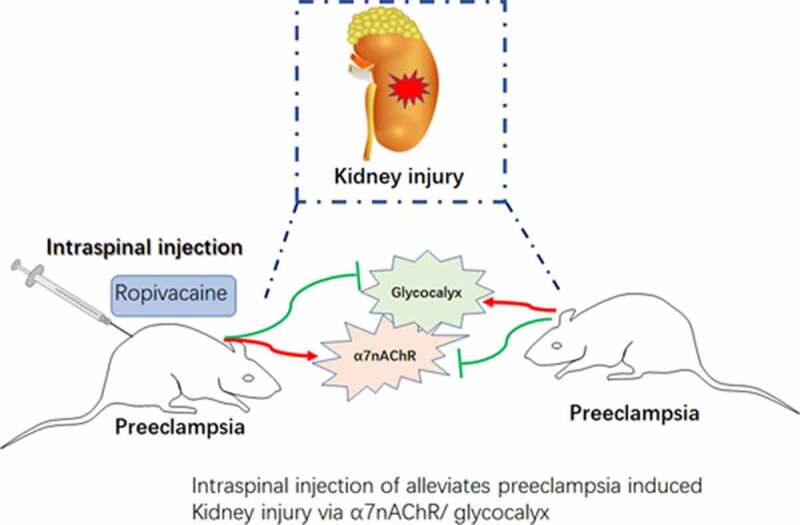

## Highlights

- Ropivacaine via intraspinal administration suppresses α 7nAChR level
- α 7nAChR/ glycocalyx involved in anti-inflammatory pathway
- α 7nAChR/ glycocalyx associated with preeclampsia induced Kidney injury

## Introduction

Preeclampsia is a sudden increase in blood pressure and/or swelling of the face and limbs during pregnancy, often accompanied by proteinuria [1]. Preeclampsia is the most common complication during pregnancy and usually develops in the second trimester (after 20 weeks), with an overall incidence of about 5% of pregnant women [2]. In recent years, Preeclampsia has fewer complications due to the popularity of prenatal examination and specific intervention, but there are still some patients with preeclampsia can develop into serious eclampsia, performance in pregnant women to severe convulsions, coma, and even death, which are serious damage to the mother and the fetus health even life [3]. The main characteristics of preeclampsia are hypertension and proteinuria in the middle and late stages of pregnancy, often accompanied by acute renal failure and preeclampsia nephropathy, etc. Kidney injury sometimes was suddenly aggravated in the late stages of pregnancy or even after delivery, but there is no effective prevention and control method to intervene at present.

The nervous and immune systems interact in complicated ways to maintain homeostasis and respond to stress or injury, and rapid nerve conduction can provide instantaneous input for regulating inflammation. Inflammatory reflection, also known as the cholinergic anti-inflammatory pathway, can adjust the innate and adaptive immunity, vagus nerve stimulation (VNS), and nicotine by α7nAChR to adjust the reflection and play anti-inflammatory effects in a variety of inflammatory diseases, such as rheumatoid arthritis, inflammatory bowel disease, and Alzheimer’s, Parkinson’s disease [4]. Some previous studies have suggested that α7nAChR mediated cholinergic anti-inflammatory pathway may be involved in the pathogenesis of preeclampsia [5,6]. Inoue T et al. showed that VNS can reduce inflammatory response by activating α7nAChR, thus playing a protective role in renal ischemia reperfusion injury [7]. In another animal study, the researchers found that C1 neurons in the medulla oblongata can be mediated adaptability-independent reaction to physiological stress, such as low blood pressure, and bleeding, by activating α7nAChR cholinergic anti-inflammatory pathway to protect the kidney from ischemia-reperfusion injury in mice [8]. Previous studies have demonstrated that the incidence of acute kidney injury (AKI) increases after orthopedic surgery [9,10], and spinal anesthesia significantly reduces the risk of postoperative AKI along with shortening the length of hospital stay [11]. A retrospective analysis of 1540 patients undergoing abdominal aortic aneurysm repair showed that patients with epidural combined with general anesthesia had lower dialysis requirements than those with general anesthesia alone [12]. Therefore, intraspinal anesthesia may play a role in reducing renal injury.

Abnormal levels of vascular endothelial factor and coagulation dysfunction are responsible for the clinical manifestations of preeclampsia, and all damages to its target organ tissue can be jointly attributed to endothelial injury. Glomerular endothelial injury in preeclampsia leads to renal injury and proteinuria progression [13]. In patients at high risk of cardiovascular disease including diabetes and hypertension, endothelial injury can lead to the development of kidney disease and cardiovascular disease, the key mechanism of which is glycocalyx (GCX) degradation, which leads to proteinuria and kidney damage. It has been shown that GCX can be regarded as a therapeutic target to prevent the progression of renal and cardiovascular diseases because of its reversible dysfunction [14].

At present, the most common and effective method for labor analgesia is intraspinal block. Our previous study indicated that intraspinal injection of ropivacaine in pregnant women with preeclampsia can reduce their blood pressure. Compared with the control group, Syndecan-1 and heparin sulfate (HS) in maternal serum were significantly decreased, and α7nAChR expression in placental tissue was significantly up-regulated as well as the symptoms of proteinuria in puerpera were also relieved [15,16]. The protective effect of intraspinal labor analgesia may be related to spinal canal block to inhibit systemic inflammatory response and protect GCX from injury by enhancing vagal nerve activity and activating cholinergic anti-inflammatory pathway [17]. Based on the previous studies, we hypothesized that Ropivacaine via intraspinal administration might affect α7nAChR/glycocalyx to be conducive to kidney injury induced by preeclampsia. In the present study, the blood pressure of preeclampsia pregnant rats treated with ropivacaine in the spinal canal was measured, the renal function was detected by biochemical instrument, and the expression and location of α7nAChR, GCX, and other cholinergic anti-inflammatory pathway molecules in kidney tissues were detected by immunohistochemistry. We purposed to explore the protective effect and the mechanism of intraspinal analgesia on maternal kidney injury in preeclampsia to provide a theoretical basis for the prevention and treatment of kidney injury in preeclampsia.

## Materials and methods

### Drug, reagent, and antibodies

Endotoxin (E8029-1VL) and ropivacaine (B5274) were purchased from Sigma-Aldrich Corp., St. Louis (MO, USA). Antibodies of α7nAChR (ab10096), Syndecan-1 (ab128936) were purchased from Abcam (UK). Elisa kits for Syndecan-1 (JL20972) and HS (JL47241) were purchased from Jiang Lai Biological Company (China). TUNEL staining kit (KY1003) was purchased from Shanghai Yuanke Biotechnology Co., LTD.

### Animal models and treatment

Thirty female and male, respectively, SPF Sprague–Dawley(SD) rats aged 8–12 weeks were supplied by Shanghai Sippr-Bk Lab Animal Co., Ltd., Shanghai, China. The animals were raised in a standard environment with 12-h light/12-h dark, humidity of 55% ± 10%, 25 ± 2°C and free access to food and water. After adaptive feeding for a week, female and male rats were initially caged at a ratio of 1:1. Gestation day 1 was defined when a vaginal plug was found in female rats.

On the 14th day of gestation, the model rats were injected with endotoxin (1.0 μg/kg) in 2 mL normal saline through tail vein to establish preeclampsia in pregnant rats. Animals in the Ropivacaine treated group were given 0.75% ropivacaine 40 μ L intraspinally once a day for 5 days after endotoxin injection. The placebo control animals were injected equal volume of normal saline. Urine of rats was collected on the day 13, 15, and 19 of gestation (about 1 ml of caudal vein sample).

Urine of rats was collected on the 13th day of gestation (about 1 ml of caudal vein sample), and animals in the Ropivacaine treated group were given 0.75% ropivacaine 40 μ L intraspinally once a day for 5 days after endotoxin injection. The placebo control animals were injected equal volume of normal saline.

The systolic blood pressure (SBP) and diastolic blood pressure (DBP) on the gestation day 13, 15, 17, 19 and 21 were measured as well as 24-h urinary protein levels on the gestation day 13, 15 and 19 were detected in all pregnant rats. On gestation day 21, the kidney of the rats was collected for further detection. The studies were according to the Animal Care Committees of the Fudan University and followed the EU Directive 2010/63/EU on the protection of animals used for scientific purposes.

### SBP and urinary protein levels assay

SBP and DBP were measured using an ALC-NIBP noninvasive system (Shanghai Alcott Biotech Co., Ltd., Shanghai, China), and 24-h urinary protein was quantified using a LH750 fully automated hematology analyzer (Beckman-Coulter Inc., Brea, CA, USA).

The levels of Syndecan-1 and HS in the serum of animals in each group were assayed using a commercial ELISA kit according to the manufacture’s instruction [16].

### TUNEL staining

Paraffin sections were prepared according to the general procedure [18]. Then, the sections were dewaxed, repaired, and slightly dried successively. After film breaking, the sections were incubated with reagents as mix appropriate reagents 1 (TdT) and 2 (dUTP) in the TUNEL kit (2:29), undergoing DAB stained, re-stained the nucleus with hematoxylin, and dehydrated using gradient concentration of ethanol in turn. Finally, sections were observed under an inverted microscope and images were collected.

### Hematoxylin-eosin staining

Paraffin sections were stained using hematoxylin-eosin according to the general protocol [19]. Pictures were collected using a microscope.

### Immunohistochemistry

Paraffin sections were dewaxed to water, antigenic repaired, endogenous peroxidase blocked, bovine serum albumin sealed and then co-cultured with primary antibody followed by incubation with secondary antibody [20]. DAB was used to color the slices and hematoxylin was used to re-stain the nuclei. Afterward, the slices were dehydration sealed and images were collected by microscope photography system.

### Data analyzed

The measurement data were analyzed using GraphPad Prism 9 software (USA) and are denoted by mean ± standard deviation (SD). The average optical density (AOD) was acquired by ImageJ software. Comparison between the two groups was performed using the unpaired T-test. Comparisons of three or more groups were performed using one-way ANOVA with proper post-hoc test. The difference was statistically significant when the p value was less than 0.05.

## Results

As one of the most common complications during pregnancy, preeclampsia could induce kidney injury. Glycocalyx mediated endothelial injury may result in kidney damage and α7nAChR correlates with stress or injury. Our previous study showed that ropivacaine with intraspinal injection attenuated preeclampsia-induced hypertension and proteinuria. In the present study, we aimed to investigate the role of intraspinal analgesia with ropivacaine in preeclampsia-induced kidney injury and GCX/α7nAChR involved. Preeclampsia model of rats was made and with intraspinal injection of Ropivacaine. Blood pressure and albuminuria were measured, pathological analysis and cell apoptosis in renal tissue were performed, α7nAChR, GCX markers as Syndecan-1 and HS were detected.

### Ropivacaine alleviates preeclampsia mediated kidney injury and high blood pressure

**Figure 1.**
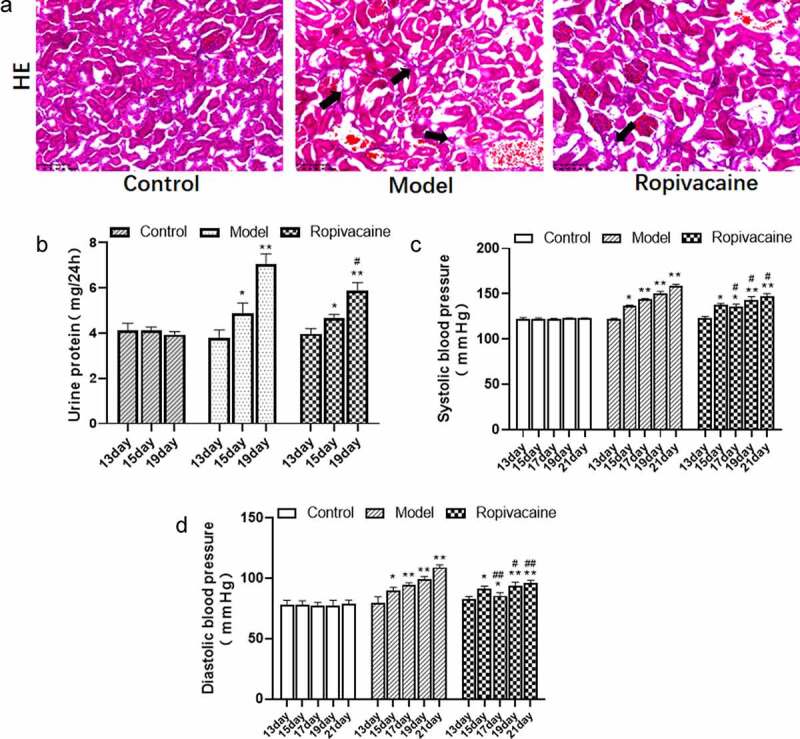
Pregnancy rats injected with normal saline were in the control group (Control) and those were injected with endotoxin to induce preeclampsia model (Model). The rats with preeclampsia were treated with ropivacaine intraspinally (Ropivacaine). (a) HE staining was performed in the kidney of rats for pathology. The arrow indicated renal tubules atrophy and inflammatory infiltration. (b) 24-h urinary protein levels on the gestation day 13, 15, and 19 were detected in all pregnant rats. (c) The systolic blood pressure (SBP) on the gestation day 13, 15, 17, 19, and 21 were measured. (d) The Diastolic blood pressure(DBP) on the gestation day 13, 15, 17, 19, and 21 were measured. *P < 0.05, **P < 0.01 compared with Control; #*P* < 0.05, ##*P* < 0.01 compared with Model. n = 5.

To investigate the effect of Ropivacaine through intrathecal injection on endotoxin-induced preeclampsia in rats, HE staining and urinary protein as well as blood pressure measures were performed. As shown in Figure 1a, significant renal tubule atrophy, endothelial cell disorder, and inflammatory infiltration were observed in model group compared with Control. Animals treated with Ropivacaine showed palliative pathological features obviously. It was similar to the results of urine protein assay that the levels of urine protein in model group were increased on both day 15 and day 19 (Figure 1b). Moreover, Ropivacaine decreased urine protein after 1 and 5 days of treatment. As high blood pressure is a typically pathological feature of preeclampsia, we also measured the blood of all the rats, results of which indicated that Ropivacaine has the effect of lowering blood pressure of SBP and DBP after 3, 5 and 7 days of treatment (Figure 1c-d).

### Ropivacaine suppresses cell apoptosis in kidney of preeclampsia rats

**Figure 2.**
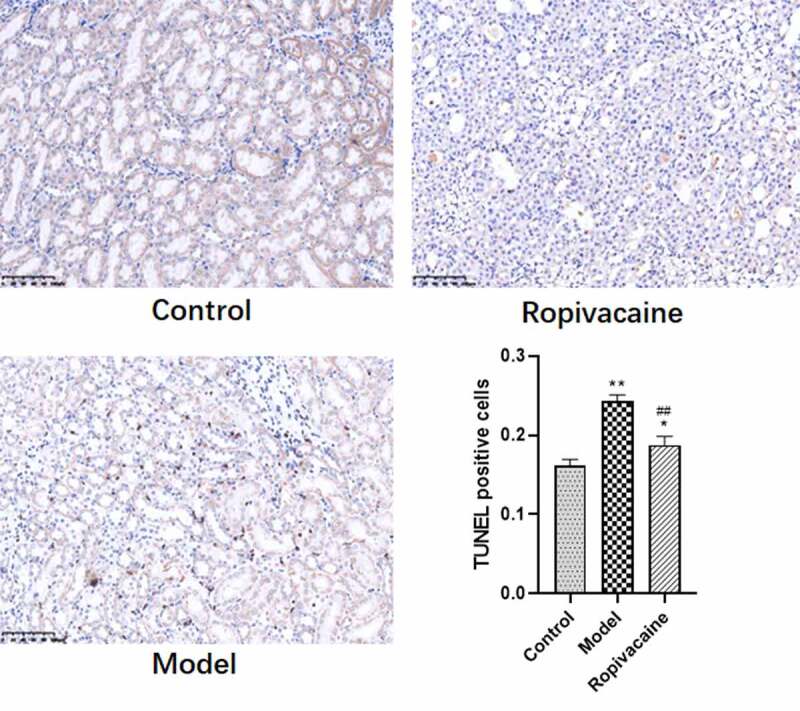
Pregnancy rats injected with normal saline were in the control group (Control) and those were injected with endotoxin to induce preeclampsia model (Model). The rats with preeclampsia were treated with ropivacaine intraspinally (Ropivacaine). Apoptosis of cell in the kidney of all the rats was assayed by TUNEL staining. *P < 0.05, **P < 0.01 compared with Control; ##*P* < 0.01 compared with Model. n = 5.

To explore the effect of Ropivacaine on preeclampsia mediated the kidney injury, TUNEL staining to measure apoptosis in the kidney of rats was performed in the followed study. Results are shown in Figure 2 that increasing apoptosis cells were observed in preeclampsia model rats, which were inhibited by Ropivacaine treatment significantly (Figure 2).

### Ropivacaine decreased Syndecan-1 and HS levels in serum of preeclampsia rats

**Figure 3.**
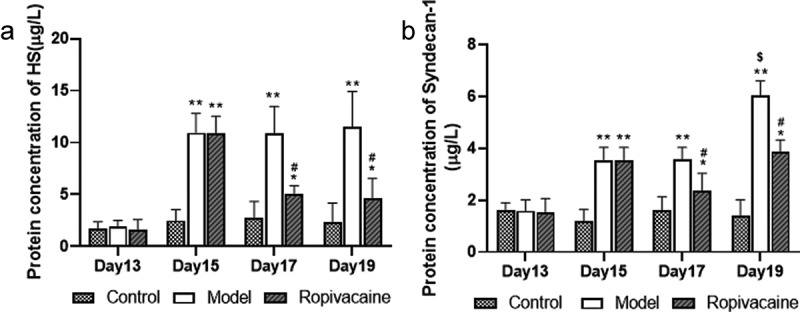
Pregnancy rats injected with normal saline were in the control group (Control) and those were injected with endotoxin to induce preeclampsia model (Model). The rats with preeclampsia were treated with ropivacaine intraspinally (Ropivacaine). The serum of rats on the gestation day 13, 15, 17, and 19 was collected. (a) The level of HS in the serum was assayed using ELISA kit. (b) The level of Syndecan-1 in the serum was assayed using ELISA kits. *P < 0.05, **P < 0.01 compared with Control; #*P* < 0.05 compared with Model. $ *P* < 0.05 compared with model on the gestation day 17. n = 5.

Syndecan-1 families are anchored on the endothelial cell membrane and can bind to glycosaminoglycan chains, which form Proteoglycans, through specific binding sites [21]. Heparin sulfate (HS) is the most common glycosaminoglycan chains [22,23]. We then measured the level of Syndecan-1 and HS in the serum of preeclampsia rats. The level of HS was significantly increasing in preeclampsia model rats induced by endotoxin on the gestation day 15, 17 and 19 (*P* < 0.01). Ropivacaine suppressed HS level obviously after 3 and 5 days of treatment (Figure 3a). Results of Syndecan-1 assay were similar to HS that preeclampsia rats have higher level of Syndecan-1 in serum 1, 3, and 5 days after model made which was decreased by Ropivacaine significantly after 3 and 5 days of treatment (*P* < 0.05) (Figure 3b). The results of HS and Syndecan-1 detection suggested that glycocalyx mediated vascular endothelial cell injury occurred in preeclampsia model rats which were attenuated by Ropivacaine.

### Ropivacaine regulates α7nAChR and Syndecan-1 expression in kidney of preeclampsia rats

**Figure 4.**
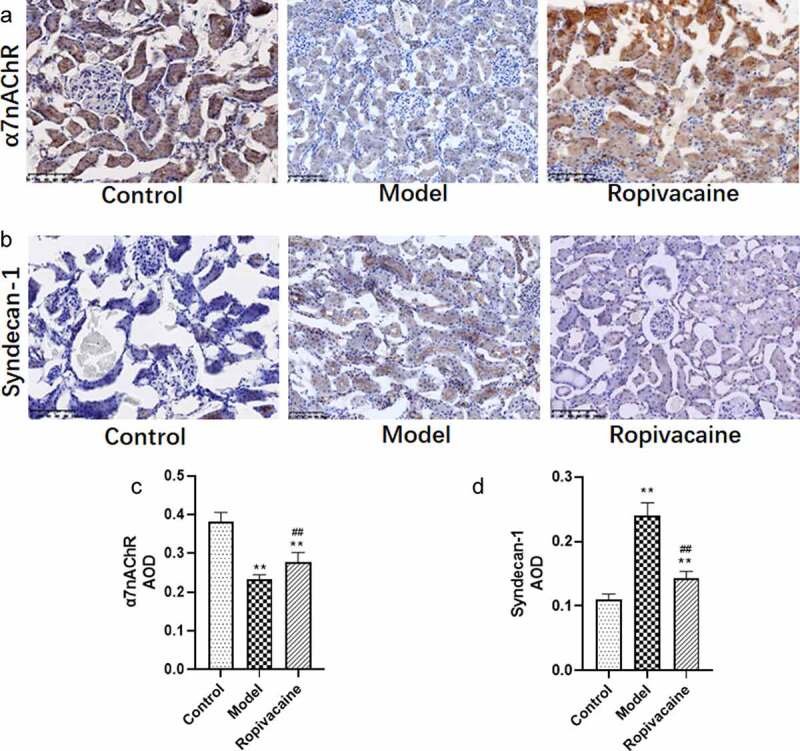
Pregnancy rats injected with normal saline were in the control group (Control) and those were injected with endotoxin to induce preeclampsia model (Model). The rats with preeclampsia were treated with ropivacaine intraspinally (Ropivacaine). The kidney of rats on the gestation day 21 was collected. (a) Protein expression of α7nAChR in the kidney was detected using immunohistochemical staining. (b) Syndecan-1 expression was measured using immunohistochemical staining. (c)Average optical density of α7nAChR assayed using immunohistochemical staining. (d) Average optical density of Syndecan-1 assayed using immunohistochemical staining. **P < 0.01 compared with Control; ##P < 0.01 compared with Model. n = 5.

As reported in previous studies, α7nAChR mediated cholinergic anti-inflammatory pathway may be involved in the pathogenesis of preeclampsia [5,6], the expression of α7nAChR was measured in the kidney of preeclampsia rats. As shown in (Figure 4a,c), the expression of α7nAChR was significantly down-regulated in the kidney of preeclampsia rats compared with that in control group. Ropivacaine suppressed induction by preeclampsia to α7nAChR expression on gestation day 21.

Syndecan-1 expression in the kidney of rats was further measured, the results of which were consistent with serum Syndecan-1 level that preeclampsia rats have much higher Syndecan-1 than control animals. The expression of Syndecan-1 in the kidney of preeclampsia rats treated with Ropivacaine was downregulated dramatically (Figure 4b,d). It may be suggested that Ropivacaine could upregulate α7nAChR while downregulate Syndecan-1 expression in the kidney of preeclampsia rats.

## Discussion

Proteinuria is one of the most typical pathological features of preeclampsia, which was a marked characteristic of kidney injury [1]. Acute renal failure and preeclampsia nephropathy was of frequent occurrence in preeclampsia patients. In the present study, preeclampsia-induced kidney injury was explored. Our results indicated that endotoxin-induced preeclampsia rats showed hypertension and higher levels of proteinuria than control animals. Cell apoptosis in the kidney of preeclampsia rats was further explored in the present study, results of which indicated that the rate of apoptosis in the kidney of model rats was significantly increased compared with that in control rats. Our results confirmed that kidney injury occurred in preeclampsia model animals.

GCX is a glycoprotein complex that covers the surface of vascular endothelial cells and is an important component of vascular endothelial barrier [24,25]. The GCX skeleton structure is composed of Proteoglycans, which is formed by one or more glycosaminoglycan chains (GAG) [26] by covalent bonding with the core protein of the endothelial cell membrane. Phosphatidylinositol proteoglycan (Glypican) and Syndecan-1 families anchored on the endothelial cell membrane belong to the core proteins of GCX [27]. Syndecan-1 can be divided into extracellular segment, intracellular segment, and transmembrane domain, and its extracellular segment can be bind to GAG through specific binding sites [21]. Chondroitin sulfate, HS, and hyaluronic acid (HA) are the most common GAG [22,23], and of which HS accounts for about 50% ~ 90%. Therefore, syndecan-1, HS, hyaluronic acid shedding in blood and changes in the thickness of GCX can be used to determine the level of GCX in endothelial cells. A previous study indicated that endothelial injury plays an important role in kidney injury. As endothelial cells at the base of the glomerulus, podocytes injury is closely related to renal-injury-induced proteinuria and high proteinuria is one of the main features of preeclampsia. Changes in podocyte structure and function are related to glomerular filtration barrier pathology, and currently become the diagnostic target of glomerular lesions [14]. Endothelial injury can lead to the development of kidney disease and cardiovascular disease in patients with diabetes and hypertension [28,29], Progress of endothelial injury is mainly due to GCX degradation, which leads to proteinuria and kidney damage. We then measured the levels of Syndecan-1 and HS in the serum of rats. The results showed that the levels of both Syndecan-1 and HS were remarkably rose in serum of preeclampsia rats compared with normal animals. The expression of Syndecan-1 in kidney of preeclampsia rats was found upregulated significantly in the present study. Our results further verified that GCX indicated by Syndecan-1 and HS was significantly increased accompanied with kidney injury induced by preeclampsia.

α7nAChR mediates the anti-inflammatory effects of cholinergic stimulation. Yeboah et al. reported that α7nAChR was expressed functionally in the tubular epithelial cells [30]. Moreover, the ischemia-reperfusion (I/R) injury induced upregulated expression of α7nAChR in the endothelium of cortical peritubular capillaries of kidney, which could be suppressed by nicotine to improve the outcome after renal I/R injury [30]. It was reported in another study that α7nAChR in macrophages was activated by acetylcholine released from the vagus nerve subjected to stimulation, while vagus nerve stimulation reduced renal I/R injury [31]. α7nAChR could be activated by Dexmedetomidine to effectively alleviate Lipopolysaccharide-induced kidney injury in mice [32]. In our study, the expression of α7nAChR in the kidney of preeclampsia rats, results of which showed that renal α7nAChR expression was downregulated in preeclampsia rats compared with normal animals. Our results were similar to the previous study that α7nAChR expression was down-regulated in the kidney induced by Lipopolysaccharide [33].

It has been shown in previous studies that the incidence of acute kidney injury (AKI) increases after orthopedic surgery [9,10], and spinal anesthesia significantly reduces the risk of postoperative AKI and shortens hospital stay period [11]. A retrospective analysis of patients undergoing abdominal aortic aneurysm repair showed that patients with epidural combined with general anesthesia had lower dialysis requirements than those with general anesthesia alone [12]. Therefore, spinal anesthesia, including epidural anesthesia and spinal anesthesia, may have a role to play in reducing renal injury. Inhalation anesthetics such as sevoflurane and enflurane have protective effects on the kidney [29]. Animal experiments have confirmed that propofol can reduce markers of oxidative stress in the kidney. Dexmedetomidine can enhance renal blood flow and glomerular filtration rate by reducing the secretion of vasopressin, thus exerting a protective effect on the kidney [34,35]. A clinical study in patients with heart valve surgery in the randomized controlled trial showed that propofol treatment group has less AKI [36]. In addition, α7nAChR and its involved cholinergic anti-inflammatory pathway connect the nervous system and immune response system through the vagus nerve. For instance, α7nAChR was triggered by electroacupuncture in the spinal cord [37]. In our previous studies, α7nAChR expression in placental tissue was significantly up-regulated in the group with intraspinal labor analgesia of ropivacaine injection [15,16]. Therefore, the effect of ropivacaine of intraspinal analgesia in kidney injury mediated by preeclampsia was explored in the present study. The expression of α7nAChR in the kidney of preeclampsia rats treated with ropivacaine in the spinal canal was significantly increased compared with those in solvent control group. It was indicated in our study that ropivacaine administrated in the spinal canal alleviated proteinuria, hypertension, apoptosis of renal cells and suppressed GCX but activated α7nAChR mediated cholinergic anti-inflammatory pathway in preeclampsia rats.

## Conclusion

In conclusion, our results suggested that Ropivacaine administered through the spinal canal may protect preeclampsia-induced renal injury by decreasing GCX and activating α7nAChR expression. α7nAChR involved cholinergic anti-inflammatory pathway and antagonists along with agonists of α7nAChR would be explored in our further study.

## Supplementary Material

Supplemental Material

## Data Availability

All data could be available from the corresponding author.
