# Supplementary material for: Ropivacaine with intraspinal administration alleviates preeclampsia-induced kidney injury via glycocalyx /alpha 7 nicotinic acetylcholine receptor pathway
Source: Bioengineered. 2022 May 29;13(5):13131–40. doi: 10.1080/21655979.2022.2080365 (PMC9275932; doi:10.1080/21655979.2022.2080365)
Supplement: Supplemental Material [file KBIE_A_2080365_SM8074.zip › Ethical approvement.pdf]

## 复旦大学实验动物科学部

## 动物实验项目伦理审查批件

| 项目名称 | **椎管内注射罗哌卡因对子痫前期产妇肾损伤的预防及保护作用研究** |
| --- | --- |
| 项目负责人 | 孙申 |
| 所属单位 | 复旦大学附属妇产科医院 |
| 通过该项目的动物实验方案，批准号：**202103029S**<br><br>复旦大学实验动物科学部动物福利和伦理小组（盖章）<br><br>日期：2021 年 3 月 30 日 | |
| 备注 | 本批件仅可用于**科研项目申报、立项及动物实验开展**之需，作他用或涂改批件一律无效。 |

# 复旦大学实验动物科学部动物实验伦理审查申请表

申请号：____________________（根据邮件回复填写）

申请原因：□项目申报 ■论文发表 □成果报奖 □其他（请注明）__________

| 一、项目基本信息 | |
|---|---|
| 项目状态 | □申请中 ■实施中 □已完成 |
| 动物实验进展 | ■尚未开始 □正在进行 □已经完成 |
| 项目名称 | 椎管内注射罗哌卡因对子痫前期产妇肾损伤的预防及保护作用研究 |
| 项目编号 | 20Y11907500 |
| 项目来源（请填写资助单位） | ■纵向（中央/地方财政资金资助）上海市科委科技创新行动计划医学创新研究专项<br>□横向（社会资金资助） |
| 项目起止日期 | 2020 年 12 月 1 日 至 2023 年 11 月 30 日 |
| 动物实验日期 | 年 月 日 至 年 月 日 |

| 二、项目负责人信息 | | | |
|---|---|---|---|
| 姓名 | 孙申 | 单位（科室） | 复旦大学附属妇产科医院麻醉科 |
| 技术职称 | 副主任医师 | 固定电话 | 15800391773 |
| 移动电话 | 15800391773 | 电子邮件 | sunshen1980@126.com |

| 三、动物实验操作者信息（参与活体动物实验操作的人员均需填写，超过三人请自行加行） | | | | |
|---|---|---|---|---|
| 姓名 | 单位（科室） | 技术职称 | 本实验中承担的操作 | 相关动物实验操作培训记录 |
| | | | | |
| | | | | |
| | | | | |

| 四、联系人（由项目负责人指定，或为负责人本人） | | | |
|---|---|---|---|
| 姓名 | 孙申 | 电子邮件 | sunshen1980@126.com |
| 移动电话 | 15800391773 | 固定电话 | 15800391773 |

| 五、动物实验概况 | |
|---|---|
| 本动物实验的目的和必要性 | 探讨子痫前期肾损伤与胆碱能抗炎通路分子的机制 |
| 动物实验内容（方案概况和观察指标） | SPF 级 SD 雌性/雄性大鼠各半。先将雌鼠与雄鼠按 1：1 的比例合笼，发现雌鼠阴栓记为妊娠第 1 天并编号，持续观察腹部膨隆确定为妊娠。大鼠妊娠第 13 天尾静脉抽血 3-5 ml，留样用于 Elisa 测定各指标基础值。测量收缩压并进行 24 h 尿蛋白定量、肌酐、尿素氮检测。将极低剂量内毒素（1.0 μg/kg，sigma）用生理盐水稀释至 2 ml，在大鼠妊娠第 14 天使用微量注射泵由尾静脉缓慢泵入，建立符合子痫前期诊断标准的子痫前期孕鼠的动物模型。 |
| 动物用量确定依据（具体到每组用途及数量） | 在大鼠妊娠第 15 天将子痫前期模型孕鼠随机分为 5 组：Control-H：模型动物皮下注射生理盐水组（n=10）；Control -IT：模型动物鞘内注射生理盐水组（n=10）；Nicotine-H：模型动物皮下注射烟碱组（1mg/kg/d）（n=10）；Ropivacaine -IT：模型动物鞘内注射盐酸罗哌卡因组（n=10）；Silver snake toxin-H：模型动物皮下注射 α -银环蛇毒素组（1μg/kg/d）（n=10）。根据预实验的结果，鞘内罗哌卡因给药 8 小时后阻滞效果基本消退，故选择每八小时给药一次。皮下注射 α 7nAChR 的特异拮抗剂 α -银环蛇毒素作为阴性对照；皮下注射 α 7nAChR 激动剂烟碱作为阳性对照。 |
| 疾病动物模型 | □不涉及 ■涉及（动物模型名称）：子痫前期 |
| 动物实验类别 | ■常规（不具备感染性、放射性、化学危害或其他生物危害性）<br>□感染性（所用感染物质）无<br>□化学毒性（所用化学物质）无<br>□放射性（所用放射性物质）无<br>□其他（请注明）无 |
| 动物尸体处理 | ■委托动物实验设施所属单位统一处理 □自行处理（请说明） |
| 涉及动物福利伦理的其他问题或对审查的特殊要求 | （没有请填“无”） 无 |
| 附件清单 | |

| 六、动物实验详情 | |
|---|---|
| 是否基因修饰动物 | ■否 □是（请说明修饰基因名称及其对动物健康和生活质量的影响） |
| 动物品种 | ■大鼠 □小鼠 □豚鼠 □地鼠 □兔 □犬 □猕猴 □小型猪<br>□其他（请说明） |
| 动物品系 | |
| 动物等级 | □普通级 □清洁级 ■SPF 级 □无菌动物 □悉生动物 □其他（请说明） |
| 动物来源 | □采购 供应商 上海斯莱克实验动物有限责任公司 生产许可证号 SCXK（沪）2017-0005 |

| | |
|---|---|
| | ☐实验室自繁 实验室所属单位<br>☐赠与 赠与单位和赠与人 |
| 动物实验设施 | 1、设施单位： 使用许可证号<br>2、设施设备类别： ☐单纯屏障设施 ☐屏障设施+IVC ☐普通设施 ☐普通设施+IVC ☐隔离器<br>☐其他（请说明） |
| 饲养管理 | 1、动物到达实验室开始实验前：观察和适应性饲养 7 天<br>2、实验期间：每笼 1 只；■常规饲养 ☐特殊饲养（请注明） |
| 实验终点（可多选，按需文字说明） | ■动物死亡<br>☐特定临床症状/体征：<br>■固定时间点： 分娩<br>☐特定实验室指标：<br>☐其他： |
| 实验操作（本栏仅供选择操作项目，每项操作均请按提示以附件形式提交详细步骤） | 1、给药（附件说明：药物名称，确切给药部位，给予体积/次.只，频率，次数等）<br>☐灌胃 ☐皮下注射 ☐腹腔注射 ☐肌内注射 ☐静脉注射 ☐皮内注射<br>☐其他注射途径（请说明） 鞘内注射<br><br>2、采血（附件说明：采血部位和方法，是否终末采血，是否麻醉，一次/24h 累计采血量，频率，次数等）<br>☐耳静脉采血 ☐尾静脉采血 ☐前肢静脉采血 ☐后肢静脉采血 ■眼底穿刺采血<br>☐其他途径和方法（请说明）<br><br>3、采集活体组织（附件说明具体操作）<br>☐尾尖 ☐ 脚趾 ☐肝脏 ☐骨髓<br>☐其他组织（请说明） 肾脏<br><br>4、手术（附件说明：手术名称，主要步骤，麻醉、镇痛药物及方法，术后护理）<br>☐终末性手术（术后不要求动物复苏）<br>■存活性小手术（不需打开胸腔、腹腔或颅腔，或造成永久肢体损伤，且术后要求动物复苏）<br>☐存活性大手术（需要打开胸腔、腹腔或颅腔，或造成永久肢体损伤，且术后要求动物复苏）<br><br>5、为辅助实验操作而进行饮食和身体限制（附件说明具体操作和目的及如何监控实施过程）<br>☐禁食 ☐限饲 ☐禁水 ☐限水 ■器械保定<br><br>6、行为学项目（附件说明具体操作等）<br>☐水迷宫 ☐高架十字迷宫 ☐穿梭箱 ☐旷场<br>☐其他项目（请说明） 无<br><br>7、心理应激项目（附件说明具体操作及如何监控实施过程等）<br>☐光刺激 ☐声刺激 ☐昼夜颠倒 ☐电击 ☐制动 ☐冷刺激 ☐热刺激 ☐饮食剥夺 ☐社交剥夺<br>☐其他项目（请说明） 无<br><br>8、其他操作：请列出操作项目名称，附件逐一说明操作步骤和部位、药物及其用法、实施频率、次数等（没有请填“无”） 无 |
| 预期本实验对动物的伤害及其处理预案（没有请填“无”） | 1、疼痛/应激：■短暂或轻微的 ☐可通过适当方法缓解 ☐持续而无法缓解 ☐强烈而无法缓解<br>2、肢体残缺/功能丧失： 无<br>3、疾病/临床症状： 高血压<br>4、简述控制动物疼痛和应激的措施以及一旦动物不能继续耐受实验时的处理：<br>安乐处死 |
| 预期实验操作中的意外情况及其处理预案 | （没有请填“无”） 无 |
| 安乐死方法 | ☐二氧化碳吸入 ■颈椎脱臼 ☐迅速断头<br>☐过量麻醉（麻醉剂及其用法）<br>☐其他方法（请说明） |

以下空白
